# Association of the Geriatric Nutritional Risk Index With Prognosis in Patients With Acute Coronary Syndrome Undergoing Percutaneous Coronary Intervention

**DOI:** 10.1002/iid3.70321

**Published:** 2026-01-15

**Authors:** Yuewen Qi, Xinchen Wang, Yan Liu, Ying Zhang, Qiyu Sun, Chen Wei, Ge Song, Jingyi Liu, Fei Shi, Lixian Sun

**Affiliations:** ^1^ Hebei Key Laboratory of Panvascular Diseases Chengde China; ^2^ Central Laboratory, The Affiliated Hospital of Chengde Medical University Chengde China; ^3^ Department of Cardiology The Affiliated Hospital of Chengde Medical University Chengde China; ^4^ The Cardiovascular Research Institute of Chengde Chengde China

**Keywords:** acute coronary syndrome, geriatric nutritional risk index, major adverse cardiovascular events, percutaneous coronary intervention

## Abstract

**Background and Aims:**

The geriatric nutritional risk index (GNRI) has shown good predictive value for some diseases. However, its association with major adverse cardiovascular events (MACEs) in acute coronary syndrome (ACS) patients undergoing percutaneous coronary intervention (PCI) remains uncertain. This study investigated the correlation between the GNRI and MACEs.

**Patients and Methods:**

This was a prospective cohort study. We consecutively enrolled 1515 ACS patients who underwent PCI. The median duration of follow‐up was 1000 days. The primary endpoints were MACEs, including all‐cause mortality, severe heart failure rehospitalization, revascularization, acute myocardial infarction (AMI) recurrence, and restenosis/intrastent thrombosis.

**Results:**

ROC curve analysis revealed an area under the curve of 0.603, with a GNRI cutoff value of 110.78. Cox regression analysis indicated that lower GNRI levels were independently associated with an increased risk of MACEs, a finding supported by risk score assessments. Kaplan–Meier survival curves and log‐rank tests indicated significantly lower cumulative survival rates in patients with lower GNRI value. Lower GNRI levels were also correlated with a higher risk of rehospitalization and cardiovascular death, as confirmed by the competing risk model. These associations remained significant after adjustments (all *p* for interaction > 0.05). RCS analysis and trend tests (all *p* < 0.05) further supported these findings.

**Conclusion:**

GNRI, as an indicator of nutritional status, was correlated with the risk of MACEs in ACS patients undergoing PCI, particularly in predicting cardiac death and rehospitalization, suggesting that the GNRI level may serve as a valid indicator for predicting poor prognosis in patients with ACS undergoing PCI.

## 1 Introduction

Acute coronary syndrome (ACS), a critical subcategory of cardiovascular disease (CVD), remains a significant global health challenge owing to its high prevalence and severe outcomes. ACS is further classified as non‐ST elevation myocardial infarction (NSTEMI) and ST elevation myocardial infarction (STEMI) based on ECG findings, and unstable angina (UA), which is no biochemical evidence of myocardial infarction. In recent years, intracoronary imaging studies showing that ACS is caused by plaque erosion rather than rupture [1]. Despite medical advancements, CVD remains the leading cause of death worldwide [2]. Malnutrition is a vital factor contributing to frailty [3], and is associated with poor prognosis in patients with chronic diseases, such as cancer and renal failure [4, 5]. However, there is a little known about the relationship between malnutrition and adverse cardiovascular events in patients with ACS undergoing PCI.

The geriatric nutritional risk index (GNRI) is a simple practical tool for assessing nutritional status, combining serum albumin levels with weight‐based calculations (present/ideal body weight). It is particularly useful for managing older and vulnerable patients. In total knee arthroplasty, GNRI has guided surgical decisions [6], while in esophageal squamous cell carcinoma, lower GNRI scores have been associated with decreased overall survival [7]. Similarly, the GNRI has been used to predict surgical site infections in patients undergoing resection of malignant musculoskeletal tumors, with lower GNRI scores linked to higher infection rates [8].

The GNRI has demonstrated substantial utility in cardiovascular care. In patients with heart failure (HF) with preserved ejection fraction, GNRI was an indicator of the risk for adverse cardiovascular events and all‐cause mortality [9, 10, 11, 12]. In acute myocardial infarction (AMI) cases, GNRI findings suggested that improving nutritional status might outweigh the benefits of aggressive lipid‐lowering therapy in malnourished patients [13].

However, despite its broad application, the relationship between the GNRI and major adverse cardiovascular events (MACEs) in patients with ACS undergoing percutaneous coronary intervention (PCI) remains unclear. This study aimed to investigate the correlation between the GNRI and MACEs, especially cardiovascular mortality.

## 2 Methods

### 2.1 Study Population

In this study, 1619 patients with ACS, including UA, NSTEMI, and STEMI, who underwent PCI, were consecutively enrolled between January 2016 and December 2018 at the Department of Cardiology, The Affiliated Hospital of Chengde Medical University (Hebei, China).

All patients underwent coronary angiography and PCI performed by an experienced team of cardiologists. Inclusion criteria included (1) age ≥ 18 years; (2) ACS diagnosis, including STEMI, NSTEMI, or UA, based on the 2013 ACCF/AHA Guidelines for the Management of ST‐Elevation Myocardial Infarction and the 2014 AHA/ACC Guidelines for the Management of Patients With Non‐ST‐Elevation Acute Coronary Syndromes; and (3) coronary stenosis ≥ 50% in at least one major artery as confirmed via coronary angiography.

Exclusion criteria included (1) in‐hospital death; (2) critical structural heart disease (e.g., dilated cardiomyopathy, hypertrophic cardiomyopathy, and aortic dissection); (3) severe inflammatory or infectious disease; (4) connective tissue disease; (5) secondary coronary vasculitis; (6) severe liver or kidney disease (creatinine clearance < 15 mL/min); and (7) patients with missing data.

The study was approved by the Ethics Committee of the Affiliated Hospital of Chengde Medical University (Approval Number: CYFYLL2015006) and conducted according to the tenets of the Declaration of Helsinki. All the participants provided informed consent.

### 2.2 Calculation of GNRI

The GNRI was calculated using the following formula: GNRI = (1.489 × albumin [g/L]) + (41.7 × present/ideal body weight) [14]. The ideal weight was determined using the Lorenz equation:

- For males, height − 100 – [(height − 150)/4].
- For females, height − 100 − [(height − 150)/2.5]^17^.

### 2.3 Baseline Demographics and Clinical Characteristics

Patient demographic and clinical data were collected by a cardiovascular research team.

Hypertension was defined as a systolic blood pressure ≥ 140 mmHg and/or diastolic blood pressure ≥ 90 mmHg at rest, or a previous diagnosis of hypertension on antihypertensive therapy [15].

Type 2 diabetes mellitus was diagnosed based on diabetes symptoms with random blood glucose ≥ 11.1 mmol/L, fasting plasma glucose ≥ 7.0 mmol/L, or a 2‐h oral glucose tolerance test ≥ 11.1 mmol/L, or by at least two blood glucose measurements meeting these criteria without symptoms [16].

Dyslipidemia was defined as serum total cholesterol (TCH) ≥ 5.18 mmol/L, high‐density lipoprotein cholesterol (HDL‐C) ≤ 1.04 mmol/L, low‐density lipoprotein cholesterol (LDL‐C) ≥ 3.37 mmol/L, triglyceride (TG) ≥ 1.7 mmol/L, or a previous diagnosis of dyslipidemia requiring medication [17].

### 2.4 Follow‐Up and Endpoints

This is a prospective cohort study with a median follow‐up duration of 1000 days. Follow‐up was completed by the cardiovascular research team, and each follow‐up abided by the principle of standardization to control bias. Follow‐up data were collected via clinical visits at 1, 3, 6, and 12 months, then annually through telephone interviews and medical records.

The primary endpoint was MACEs, including all‐cause mortality, rehospitalization for severe HF (New York Heart Association Grade IV), revascularization (re‐PCI), AMI recurrence, and restenosis/intrastent thrombosis.

### 2.5 Statistical Analysis

The normality of continuous variables was validated using the Kolmogorov–Smirnov test. Normally distributed variables were expressed as mean ± standard deviation and analyzed using the *t*‐test, whereas non‐normally distributed variables were presented as medians with interquartile ranges and compared using the Mann–Whitney *U* test. Categorical variables were presented as numbers (%) and compared using the *χ* ^2^ test. Survival analysis was conducted using the Kaplan–Meier method with group comparisons using the log‐rank test. The diagnostic accuracy of GNRI was assessed using receiver operating characteristic (ROC) curves, with the optimal cutoff value determined through Youden's index (sensitivity + specificity − 1).

Patients were divided into low and high GNRI groups based on the cutoff value as mentioned above. Previous research has shown that patients with lower GNRI levels typically experience negative outcomes, whereas those with higher GNRI levels serve as standards for comparison in subsequent statistical analyses. Univariate and multivariate Cox proportional hazards models were used to examine the ability of the GNRI to predict prognostic risk.

The risk score was calculated from the multivariate model, and the dose–response relationship between the GNRI and MACEs or all‐cause mortality in patients with ACS undergoing PCI was presented using a restricted cubic spline (RCS) curve. A competing risk model was used to investigate the correlation between GNRI and risks of rehospitalization or cardiac death, with the Gray test used for group comparisons.

Subgroup analyses were performed to investigate the GNRI's prognostic accuracy across different demographics and comorbidities. Statistical analyses were performed using SPSS version 26 (SPSS Inc., Chicago, IL, USA) and R software (4.3.1). R packages “survival,” “ggplot2,” “rms” were used in this present study. All tests were two‐sided, and *p* < 0.05 was considered statistically significant.

In this study, we applied four different models:

- Model 1: unadjusted.
- Model 2: adjusted for sex, age ≥ 65 years, hypertension, cardiogenic shock, and left ventricular ejection fraction (LVEF).
- Model 3: adjusted for sex, age ≥ 65 years, dyslipidemia, hypertension, diabetes mellitus, cardioge'nic shock, STEMI, and LVEF.
- Model 4: adjusted for sex, age ≥ 65 years, dyslipidemia, hypertension, diabetes mellitus, family history of CAD, cardiogenic shock, three‐vessel disease, STEMI, left ventricular end‐diastolic diameter (LVEDD), creatinine (Cr), and LVEF.

## 3 Results

### 3.1 Patients' Baseline Characteristics

**Figure 1:**
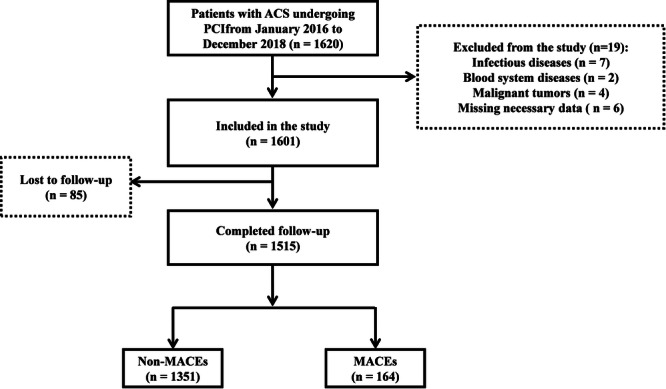
Flow chart of the study. ACS, acute coronary syndrome; MACEs, major adverse cardiovascular events; PCI, percutaneous coronary intervention.

A total of 19 patients were excluded based on the exclusion criteria, including 7 with infectious diseases, 2 with blood disorders, 4 with malignant tumors, and 6 with missing necessary data. In total, 85 patients were lost to follow‐up. The median duration of follow‐up was 1000 days. Finally, 1515 patients who completed the follow‐up were included in the final analysis. Of these, 164 (10.8%) experienced MACEs, including 52 deaths (30 cardiac‐related deaths), 4 patients with severe HF requiring rehospitalization, 28 patients with recurrent nonfatal MIs, 5 patients with stent restenosis, and 75 patients who underwent re‐PCI (Figure 1).

The present study found that UA, history of HF, cardiogenic shock, aspirin, ticagrelor, statins, diuretics, one vessel, three vessels, LVEDD > 50 mm, white blood cell (WBC) count, hemoglobin (HGB), neutrophils, lymphocytes, albumin, Cr, and GNRI values were significantly different between the MACEs group and non‐MACEs group (all *p* < 0.05).

The AUC for GNRI was 0.603 (*p* < 0.001, 95% confidence interval [CI], 0.560–0.646). Based on Youden's index, the optimal diagnostic cutoff value for GNRI was 110.78, with a sensitivity of 72.30% and a specificity of 43.52%. All 1515 ACS patients were divided into 2 groups according to the cutoff value: the low GNRI group (*n* = 634) and the high GNRI group (*n* = 881). Table 1 shows that some variables, including age ≥ 65 years, UA, STEMI, dyslipidemia, hypertension, diabetes mellitus, LVEDD, LVEF < 40%, HGB, platelets, neutrophils, lymphocytes, TCH, TG, LDL, serum uric acid, and MACEs, have significant differences between the two groups (all *p* < 0.05).

**Table 1:** Baseline characteristics of patients in the low and high GNRI groups.

Characteristic	Overall, *N* = 1515	Low GNRI group, *N* = 634	High GNRI group, *N* = 881	*p*
Sex				0.989
Female	385 (25.4%)	161 (25.4%)	224 (25.4%)	
Male	1130 (74.6%)	473 (74.6%)	657 (74.6%)	
Age ≥ 65 years old	361 (23.8%)	110 (17.4%)	251 (28.5%)	< 0.001
Height (cm)	169 (162, 172)	170 (164, 172)	169 (162, 172)	0.030
Weight (kg)	70 (63, 80)	80 (70, 85)	65 (60, 72)	< 0.001
BMI	25.1 (23.1, 27.5)	27.7 (25.8, 29.4)	23.7 (22.0, 25.2)	< 0.001
UA	604 (39.9%)	288 (45.4%)	316 (35.9%)	< 0.001
STEMI	671 (44.3%)	251 (39.6%)	420 (47.7%)	0.002
NSTEMI	240 (15.8%)	95 (15.0%)	145 (16.5%)	0.438
Dyslipidemia	859 (56.7%)	424 (66.9%)	435 (49.4%)	< 0.001
Hypertension	888 (58.6%)	422 (66.6%)	466 (52.9%)	< 0.001
DM	386 (25.5%)	194 (30.6%)	192 (21.8%)	< 0.001
Cerebral infarction	214 (14.1%)	89 (14.0%)	125 (14.2%)	0.934
History of HF	151 (10.0%)	50 (7.9%)	101 (11.5%)	0.022
Family history of CAD	209 (13.8%)	92 (14.5%)	117 (13.3%)	0.493
Cardiogenic shock	23 (1.5%)	7 (1.1%)	16 (1.8%)	0.264
Aspirin	1493 (98.5%)	628 (99.1%)	865 (98.2%)	0.163
Clopidogrel	1191 (78.6%)	490 (77.3%)	701 (79.6%)	0.285
Ticagrelor	300 (19.8%)	138 (21.8%)	162 (18.4%)	0.104
β‐blocker	781 (51.6%)	365 (57.6%)	416 (47.2%)	< 0.001
ACEIARB	686 (45.3%)	339 (53.5%)	347 (39.4%)	< 0.001
Statins	1488 (98.2%)	625 (98.6%)	863 (98.0%)	0.365
CCB	261 (17.2%)	148 (23.3%)	113 (12.8%)	< 0.001
Diuretic	106 (7.0%)	32 (5.0%)	74 (8.4%)	0.012
Spironolactone	361 (23.8%)	128 (20.2%)	233 (26.4%)	0.005
One vessel	475 (31.4%)	200 (31.5%)	275 (31.2%)	0.891
Two vessels	484 (31.9%)	212 (33.4%)	272 (30.9%)	0.291
Three vessels	556 (36.7%)	222 (35.0%)	334 (37.9%)	0.249
GENSINI	44 (29, 66)	44 (29, 66)	44 (28, 68)	0.904
LAD (mm)	35.0 (32.0, 37.0)	35.0 (32.5, 38.0)	34.0 (31.0, 37.0)	< 0.001
LVEDD (mm)	50.0 (47.0, 54.0)	51.0 (48.0, 54.3)	50.0 (47.0, 54.0)	0.003
LVSDD (mm)	34.0 (32.0, 38.0)	34.0 (32.0, 38.0)	34.0 (31.0, 38.8)	0.443
LVEF (％)	58 (53, 64)	59 (55, 64)	58 (50, 64)	0.029
LVEDD > 50 (mm)	664 (48.4%)	289 (51.2%)	375 (46.5%)	0.082
LVEF < 40%	39 (2.8%)	10 (1.8%)	29 (3.6%)	0.046
WBC (10^9^L)	7.9 (6.3, 10.3)	7.8 (6.4, 10.1)	8.0 (6.3, 10.4)	0.340
HGB (g/L)	147 (136, 157)	151 (139, 161)	145 (135, 154)	< 0.001
PLT (10^9^L)	214 (179, 251)	218 (184, 253)	211 (177, 248)	0.019
Neutrophils (10^9^L)	5.4 (3.9, 8.0)	5.2 (3.9, 7.6)	5.6 (4.0, 8.3)	0.025
Lymphocyte (10^9^L)	1.67 (1.23, 2.27)	1.74 (1.31, 2.37)	1.60 (1.18, 2.17)	< 0.001
Monocyte (10^9^L)	0.43 (0.31, 0.57)	0.43 (0.32, 0.57)	0.43 (0.31, 0.57)	0.750
MPV (fL)	10.40 (9.80, 11.00)	10.40 (9.90, 11.00)	10.40 (9.80, 10.90)	0.142
PDW (%)	12.00 (11.00, 13.40)	12.20 (11.10, 13.50)	11.90 (10.90, 13.20)	0.005
ALB (g/L)	41.1 ± 3.6	43.5 ± 2.9	39.4 ± 3.0	< 0.001
TCH (mmol/L)	4.33 (3.69, 5.06)	4.50 (3.86, 5.29)	4.21 (3.61, 4.91)	< 0.001
TG (mmol/L)	1.60 (1.05, 2.45)	1.88 (1.29, 2.89)	1.41 (0.89, 2.10)	< 0.001
HDL‐C (mmol/L)	1.08 (0.90, 1.26)	1.06 (0.90, 1.22)	1.09 (0.90, 1.29)	0.113
LDL‐C (mmol/L)	2.31 (1.85, 2.88)	2.44 (1.94, 2.99)	2.24 (1.82, 2.82)	< 0.001
Cr (μmol/L)	67 (59, 78)	68 (60, 79)	67 (58, 78)	0.126
Serum uric acid (μmol/L)	327 (265, 384)	336 (274, 398)	318 (260, 376)	< 0.001
MACEs	164 (10.8%)	46 (7.3%)	118 (13.4%)	< 0.001
All‐cause death	52 (3.4%)	10 (1.6%)	42 (4.8%)	< 0.001
Cardiac death	30 (2.0%)	5 (0.8%)	25 (2.8%)	0.005
HF	4 (0.3%)	0 (0.0%)	4 (0.5%)	0.145
AMI recurrence	28 (1.8%)	8 (1.3%)	20 (2.3%)	0.151
Stent restenosis	5 (0.3%)	1 (0.2%)	4 (0.5%)	0.407
Re‐PCI	75 (5.0%)	27 (4.3%)	48 (5.4%)	0.292

Abbreviations: ACEI/ARB, angiotensin‐converting enzyme inhibitor/angiotensin receptor blocker; ALB, albumin; AMI, acute myocardial infarction; CAD, coronary artery disease; CCB, calcium‐channel blocker; Cr, creatine; DM, diabetes mellitus; GNRI, geriatric nutritional risk index; HDL‐C, high‐density lipoprotein cholesterol; HGB, hemoglobin; HF, heart failure; LAD, left atrial diameter; LDL‐C, low‐density lipoprotein cholesterol; LVEDD, left ventricular end‐diastolic diameter; LVEF, left ventricular ejection fraction; LVSDD, left ventricular end‐systolic diameter; MACEs, major adverse cardiovascular events; HF, heart failure; MPV, mean platelet volume; NSTEMI, non‐ST‐segment elevation myocardial infarction; PCI, percutaneous coronary intervention; PDW, platelet distribution width; PLT, platelets; STEMI, ST‐segment elevation myocardial infarction; TCH, total cholesterol; TG, triglyceride; UA, unstable angina; WBC, white blood cell.

### 3.2 Cox Proportional Hazard Model

The predictive ability of the GNRI for MACEs was assessed using a ROC curve, yielding an area under the curve of 0.603 (95% CI: 0.560–0.646, *p* < 0.001) with a cutoff value of GNRI = 110.78 with the sensitivity of 0.720, specificity of 0.435. Patients were categorized into GNRI ≤ 110.78 group and GNRI > 110.78 group, with the high GNRI group serving as the reference based on previous findings that lower GNRI levels are associated with a poorer prognosis.

The Cox proportional hazards model (Table 2) revealed that a lower GNRI level was an independent risk factor.

**Table 2:** Cox proportional hazard model for predictive factors of MACEs and all‐cause death.

Variables	Model 1	*p*	Model 2	*p*	Model 3	*p*	Model 4	*p*
	sHRs (95% CI)		sHRs (95% CI)		sHRs (95% CI)		sHRs (95% CI)	
MACEs								
GNRI ≤ 110.78	1.861 (1.323–2.616)	< 0.001	1.769 (1.228–2.550)	0.002	1.846 (1.275–2.672)	0.001	1.724 (1.187–2.503)	0.004
All‐cause death								
GNRI ≤ 110.78	2.953 (1.481–5.886)	0.002	2.136 (1.041–4.382)	0.039	2.200 (1.067–4.538)	0.033	2.089 (1.004–4.348)	0.049

Abbreviations: CI, confidence interval; GNRI, geriatric nutritional risk index; MACEs, major adverse cardiovascular events; sHRs, subhazard ratios.

For MACEs:

- Model 4: HR: 1.724 (95% CI: 1.187–2.503, *p* = 0.004).

For all‐cause death, the following results were observed:

- Model 4: HR: 2.089 (95% CI: 1.004–4.348, *p* = 0.049).

**Figure 2:**
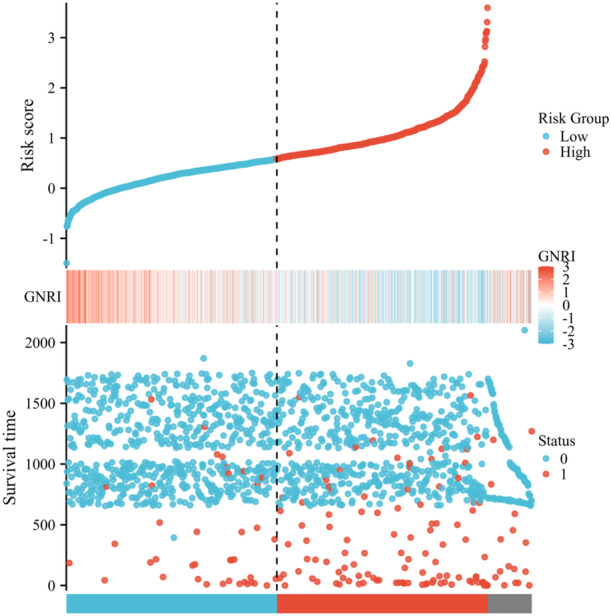
Distribution of the risk score, survival status, and expression profiles of GNRI predicting MACEs. In the heat map, red indicates higher expression levels, while light blue indicates lower expression levels. The risk scores are displayed in ascending order, with patients categorized as either low‐risk (blue) or high‐risk (red) based on a predetermined threshold (vertical black line). Each bar in the plot corresponds to the occurrence of MACEs in a specific patient. Red bars correspond to cases of MACEs, whereas blue bars indicate no recurrence during the observation period. GNRI, geriatric nutritional risk index; MACEs, major adverse cardiovascular events.

The risk scores for each patient with ACS were calculated using multivariate Cox regression analysis based on Model 4. The risk scores, survival status, and GNRI values are shown in Figure 2. The distribution of the GNRI values and risk scores in our study population was normalized using *z*‐scores. A lower GNRI score was associated with an increased risk of MACEs.

### 3.3 Kaplan–Meier Survival Curves

**Figure 3:**
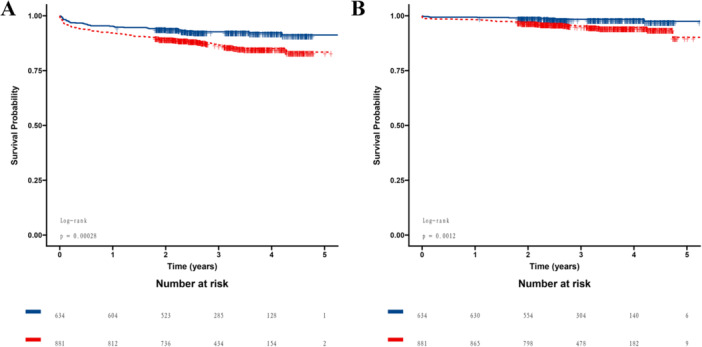
Cumulative survival probability curves of MACEs (A) and all‐cause death (B) during follow‐up stratified by the GNRI. The blue line represents the high GNRI group, and the red line represents the low GNRI group. GNRI, geriatric nutritional risk index; MACEs, major adverse cardiovascular events.

Using the GNRI cutoff, Kaplan–Meier survival curves and log‐rank test showed that patients in the low GNRI group had significantly lower cumulative survival rates than the control group (log‐rank test: *p* = 0.00028 for MACEs and *p* = 0.0012 for all‐cause death; Figure 3A,B).

### 3.4 Predicting Rehospitalization Risk and Cardiac Death Using Competing Risk Regression

A competing risk model was used to analyze the correlation between the GNRI level and rehospitalization risk, which include rehospitalization for severe heart failure HF, re‐PCI, AMI recurrence, and restenosis/intrastent thrombosis. Because all‐cause death may cause bias, it was treated as a competing event. For rehospitilization risk, the low GNRI group had a higher risk than the high GNRI group:

- Unadjusted sHRs: 1.57 (95% CI: 1.06–2.33, *p* = 0.026).
- After adjusting for age, sex, hypertension, diabetes, dyslipidemia, and cardiogenic shock, the results were sHRs: 1.68 (95% CI: 1.12–2.50, *p* = 0.011).

**Figure 4:**
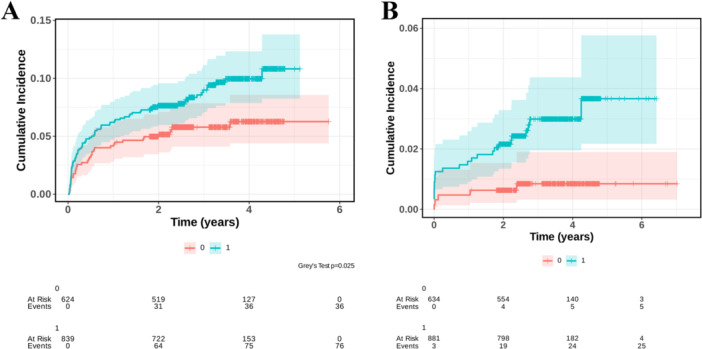
Cumulative incidence function (CIF) of the high and low GNRI groups when predicting rehospitalization risk (A) and cardiovascular death (B) by competing risk model. The red line represents the high GNRI group, and the green line represents the low GNRI group. GNRI, geriatric nutritional risk index.

The cumulative incidence function (CIF) of the low GNRI group was significantly different (Gray's test, *p* = 0.025, Figure 4A).

When predicting cardiac death, death events other than cardiogenic death are considered competitive events. For cardiovascular death, the low GNRI group showed a significantly higher risk compared to the high GNRI group:

Unadjusted sHRs: 3.56 (95% CI: 1.36–9.27, *p* = 0.009).

After adjusting for age, sex, hypertension, diabetes, dyslipidemia, and cardiogenic shock, the results were sHRs: 3.31 (95% CI: 1.19–9.20, *p* = 0.022). The CIF was significantly different between both groups (Gray's test, *p* = 0.006, Figure 4B).

### 3.5 Predictive Value of the GNRI in Various Subgroups

**Figure 5:**
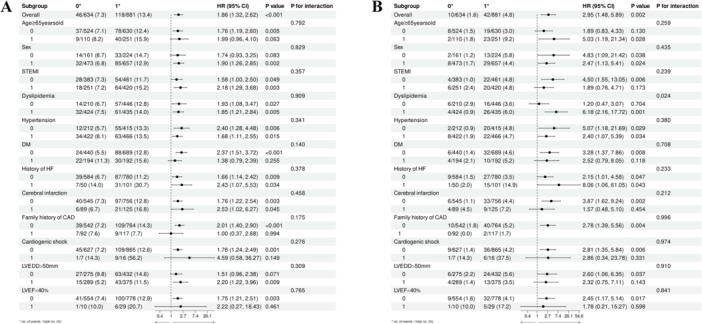
Forest plot illustrating the association of the GNRI with the risk of MACEs (A) and all‐cause death (B) in patients with ACS undergoing PCI stratified by various subgroups. ACS, acute coronary syndrome; CAD, coronary artery disease; DM, diabetes mellitus; GNRI, geriatric nutritional risk index; HF, heart failure; LVEDD, left ventricular end‐diastolic diameter; LVEF, left ventricular ejection fraction; MACEs, major adverse cardiovascular events; PCI, percutaneous coronary intervention; STEMI, ST‐segment elevation myocardial infarction.

Figure 5 shows the correlation between the GNRI value and MACEs (Figure 5A) and all‐cause death (Figure 5B) across different subgroups, including sex, age, STEMI, dyslipidemia, hypertension, diabetes, history of HF, cerebral infarction, family history of CAD, LVEF < 40%, and LVEDD > 50 mm. GNRI was a significant predictor of MACEs and overall mortality across all subgroups, with all interaction *p* values exceeding 0.05 after controlling for confounding factors.

### 3.6 Variation in GNRI and MACEs Risk by RCS and *p* for Trend

**Figure 6:**
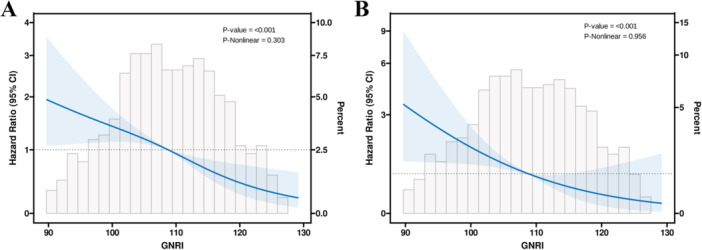
Dose–response relationship of the GNRI with the risk of MACEs (A) and all‐cause death (B) in ACS patients undergoing PCI. ACS, acute coronary syndrome; GNRI, geriatric nutritional risk index; MACEs, major adverse cardiovascular events; PCI, percutaneous coronary intervention.

RCS analysis was used to visualize the trends between GNRI as a continuous variable and adverse clinical outcomes. Figure 6A shows the relationship between GNRI and MACEs (*p* overall < 0.001, *p* nonlinear = 0.303). Figure 6B demonstrates the correlation between GNRI and overall mortality (*p* overall< 0.001 and *p* nonlinear = 0.956).

The GNRI was divided into four equal quartiles (T1–T4), with T4 serving as the reference. Cox regression analysis showed the results for MACEs, details refer to Table 3. Those results show that the GNRI level was negatively correlated with MACEs and overall mortality.

**Table 3:** Cox proportional hazard models of MACEs risk according to the GNRI subgroup.

GNRI	Unadjusted	*p*	Adjusted	*p*
	HR (95% CI)		HR (95% CI)	
T1 (≤ 103.19)	2.396 (1.471–3.903)	< 0.001	2.222 (1.313–3.759)	0.003
T2 (> 103.19 and ≤ 108.76)	1.908 (1.152–3.159)	0.012	1.737 (1.007–2.998)	0.047
T3 (> 108.76 and ≤ 114.79)	1.889 (1.139–3.135)	0.014	1.887 (1.113–3.202)	0.018
T4 (> 114.79)	1 (reference)	—	1 (reference)	—
*p* for trend		0.006		0.028

*Note:* Adjusted for: age ≥ 65 years old, STEMI, hypertension, DM, history of HF, cardiogenic shock, LVEDD > 50 (mm), and LVEF% < 40%.

Abbreviations: CI, confidence interval; GNRI, geriatric nutritional risk index; HR, hazard ratio; MACEs, major adverse cardiovascular events.

## 4 Discussion

This prospective cohort study investigated the relationship between the GNRI and MACEs, including all‐cause death, in ACS patients. We also explored the risk of rehospitalization and cardiac death using a competing risk model. The new insights of our study are as follows:

- A GNRI value ≤ 110.78 was an independent risk factor for MACEs, including all‐cause mortality.
- Lower GNRI levels were correlated with an increased risk of poor prognosis.

Additionally, lower GNRI scores remained correlated with increased risks of rehospitalization and cardiac death. Notably, this is a long‐term follow‐up study focusing on patients with ACS who underwent PCI to determine the prognostic value of GNRI.

Previous studies have demonstrated the GNRI's prognostic value across various clinical conditions. One research revealed that lower GNRI scores in patients with acute ischemic stroke had a higher incidence of stable angina pectoris [18]. Similarly, low GNRI scores have been shown to predict reduced overall survival in patients with head and neck cancer [19]. In patients with recurrent pancreatic cancer, the GNRI serves as an independent prognostic factor for survival after recurrence [20]. In addition, GNRI has been identified as an independent prognostic marker for overall and progression‐free survival in patients with large B‐cell lymphoma [21].

A meta‐analysis of cohort studies involving hemodialysis patients reinforced the association between low GNRI scores and increased risks of all‐cause and cardiovascular mortality [22]. The impact of GNRI on hospital‐related parameters has also been studied extensively. For example, in older inpatients, a lower GNRI has been linked to extended hospital stays, elevated inflammatory markers such as C‐reactive protein, and reduced lymphocyte counts [23]. Although previous research has highlighted the GNRI's prognostic value in different subgroups of CAD, our study is the first to demonstrate its correlation with increased risks of rehospitalization and cardiac death via a competing risk model.

CVD remains the leading cause of morbidity and mortality worldwide, necessitating continued research into factors that influence clinical outcomes. One area gaining attention is the nutritional status of patients and its potential prognostic significance for cardiovascular events. The GNRI, a tool combining body mass index and serum albumin levels, has emerged as a useful metric for assessing nutritional risk in older adults and at‐risk populations. A study on patients with chronic coronary total occlusion after PCI demonstrated that malnutrition, assessed using the GNRI, was an independent predictor of all‐cause mortality and MACE [24]. Malnourished patients with stable CAD exhibited higher rates of all‐cause death and MACE than their well‐nourished counterparts [25]. These findings highlight the significance of nutritional assessment in managing and predicting long‐term outcomes in patients with advanced atherosclerosis. The proposed mechanism may be associated with inflammation, metabolism, and aging [26, 27]. Additional research has explored the relationship between the GNRI scores and clinical outcomes in patients with ACS, with findings similar to those of Nakamura and colleagues, who observed that malnutrition significantly worsened long‐term mortality in older patients with AMI [28].

The relevance of the GNRI in prognostic evaluation extends beyond immediate postinterventional outcomes. Wada and colleagues assessed the long‐term impact of nutritional status in patients with CAD post‐PCI and found that lower GNRI levels independently predicted higher risks of all‐cause and cardiac mortality [29]. Comparisons with other nutritional indices reinforced the GNRI's superior predictive value. Kim and colleagues compared the GNRI with the Prognostic Nutritional Index and the Controlling Nutritional Status score in predicting post‐AMI mortality [30]. The GNRI showed a higher AUC and better incremental predictive value, suggesting it may be the most reliable among the compared indices. Additionally, Ma and colleagues demonstrated that integrating the GNRI with the GRACE score significantly improved prognostic accuracy for ACS patients undergoing PCI, particularly in diabetic subgroups [31]. Overall, the GNRI is a promising tool for assessing nutritional status, which facilitates the early identification of high‐risk individuals, enabling timely intervention and improved prognosis.

## 5 Limitations

Despite the moderate sample size and use of various innovative statistical methods, this single‐center study has several limitations. First, while we identified the GNRI as an independent risk factor for adverse outcomes in ACS patients, the specific mechanisms linking lower GNRI values to poorer prognosis in these patients remain unclear. Second, as an observational study, the findings demonstrate correlation rather than causality. Third, although we adjusted for numerous variables, the study could not control for all environmental and external factors that may have influenced patient outcomes.

## 6 Conclusion

The GNRI, a valuable indicator of nutritional status, was found to be correlated with the risk of MACEs in ACS patients undergoing PCI. Notably, the GNRI proved effective in predicting the risk of cardiac death and rehospitalization using a competing risk model. Given its simplicity and reliability in identifying high‐risk patients with ACS undergoing PCI, the GNRI holds significant promise for widespread application in clinical practice.

## Author Contributions

Study design: Yuewen Qi, Xinchen Wang, Yan Liu, Chen Wei, Ge Song, Jingyi Liu, and Lixian Sun. Acquisition of data: Xinchen Wang, Yan Liu, Ying Zhang, Chen Wei, and Yuewen Qi. Data analysis and interpretation: Xinchen Wang, Yuewen Qi, Ying Zhang, Chen Wei, Fei Shi, and Lixian Sun. Drafting and critical revision of the manuscript for important intellectual content: Yuewen Qi, Xinchen Wang, Ying Zhang, Qiyu Sun, Chen Wei, Fei Shi, and Lixian Sun. Statistical analysis: Xinchen Wang, Yuewen Qi, Yan Liu, Qiyu Sun, Wei Chen, and Lixian Sun. Supervision: Lixian Sun. All the authors contributed to the preparation of the manuscript and approved its final version.

## Ethics Statement

Ethical approval to report this case was obtained from the Institutional Review Board of The Affiliated Hospital of Chengde Medical University (Approval Number: CYFYLL2015006).

## Consent

Written informed consent was obtained from the patients.

## Conflicts of Interest

The authors declare no conflicts of interest.

## Data Availability

All raw data will be made available on reasonable request.
